# *GigaByte*: Publishing at the Speed of Research

**DOI:** 10.46471/gigabyte.1

**Published:** 2020-07-01

**Authors:** Scott C. Edmunds, Laurie Goodman

**Affiliations:** ^1^ GigaScience, BGI Hong Kong Tech Co Ltd., 26F A Kings Wing Plaza, 1 On Kwan Street, Shek Mun, Sha Tin, NT, Hong Kong, China

## Abstract

Current practices in scientific publishing are unsuitable for rapidly changing fields and for presenting updatable data sets and software tools. In this regard, and as part of our continuing pursuit of pushing scientific publishing to match the needs of modern research, we are delighted to announce the launch of *GigaByte*, an online open-access, open data journal that aims to be a new way to publish research following the software paradigm: CODE, RELEASE, FORK, UPDATE and REPEAT. Following on the success of *GigaScience* in promoting data sharing and reproducibility of research, its new sister, *GigaByte*, aims to take this even further. With a focus on short articles, using a questionnaire-style review process, and combining that with the custom built publishing infrastructure from River Valley Technologies, we now have a cutting edge, XML-first publishing platform designed specifically to make the entire publication process easier, quicker, more interactive, and better suited to the speed needed to communicate modern research.

## Future’s Past

In 2012, we launched *GigaScience* [1] as a new type of journal — one that provides standard scientific publishing linked directly to a database that hosts all the relevant data. Aiming to address Buckheit and Donoho’s 1995 complaint that “*an article about computational results is advertising, not scholarship. The actual scholarship is the full software environment, code and data that produced the result.*” [2], we were inspired to launch a new type of journal that focussed on making sure the actual scholarship of all types of research was included with the article narrative.

Eight years on we have achieved many of these aims. With data publishing becoming more mainstream as the major publishers have followed our lead, and the journal receiving awards (e.g. the 2018 Prose Awards Winner for “Innovation in Journal Publishing”) [3] and other forms of recognition for our past efforts, we are hungry to do more. One of our biggest frustrations and a major component of what has held us back has been the legacy publishing infrastructure, which, for the most part, still incorporates print-based production processes, and has a decades old codebase. It is no longer fit for purpose in this non-print, online, more data-centric digital age. When carrying out collaborations and integrations with the growing ecosystem of open science platforms, trying to make changes in scientific publishing always had to be *ad hoc* and shoehorned into legacy publishing workflows. Any technical changes and updates to the system are typically slow and buggy, and have issues with scalability. More, making changes is very expensive, and thus, to make business sense, changes have to be useful for a wide number of journals — all with different needs. Modern, web-literate platforms dominate other fields, so when we are publishing modern, web-literate research outputs, we should publish them in the same manner. The vast majority of publishing platforms remain highly focussed on static, hand-typeset PDFs rather than modern, digital first, XML- (Extensible Markup Language) based technologies.

An inspirational spark came to us from *GigaScience* Editorial Board Member Carole Goble, who won the FORCE11 Vision Prize in 2013 with her proposal “Don’t Publish. Release!” [4]. She proposed applying a 21st century software-release-style paradigm instead of an 18th century print-a-book paradigm to scholarly communication. And thus the germ of the idea that would eventually spawn *GigaByte* was set in motion.

**Figure 1.**
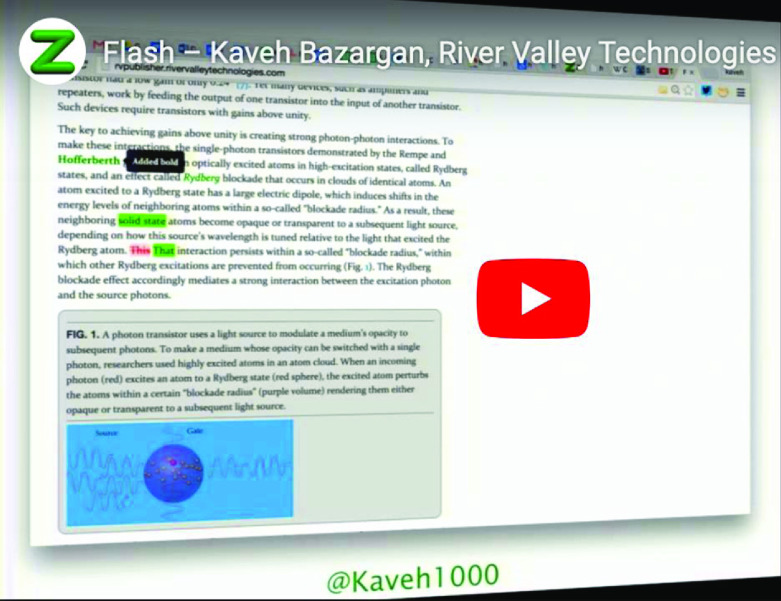
Recording and slides of Kaveh Bazargan’s talk “Why are we so attached to attachments? Let’s ditch them and improve publishing”, presented in the “Vision” track at FORCE2015 in Oxford on 13th January 2015 https://youtu.be/aFzRVqTNi-8

Among the winners of the 2015 FORCE11 Vision Prize was Kaveh Bazargan from River Valley Technologies who presented “Why are we so Attached to Attachments? Let’s Ditch Them and Improve Publishing” [5]. In this Kaveh presented embryonic ideas that would eventually becoming RVT’s end-to-end publishing solution, which includes manuscript submission, content management, and hosting using collaborative online platforms (Figure 1). Sensing this was exactly what we needed to meet our open science objectives, it sparked a collaboration to develop a publishing process that, in addition to providing on-the-fly article production, would create more interactive articles that can be versioned and forked, thereby following the “Don’t Publish. Release!” concept.

Our collaboration with RVT, with new publishing strategies and technologies, aims to address as many of these bottlenecks as possible — and, moving forward, allows us to evolve, fine tune, and reduce publishing time and cost without all the heavy lifting needed to change current publishing processes.

## Three, Two, One, Launch!

For *GigaByte*, we are launching with two article types: *Data Release* and *Technical Release* articles, focussed respectively on datasets and software/computational workflow papers, as these are the most obvious Research Objects that require the iterative form of the CODE, RELEASE, FORK, UPDATE and REPEAT approach. The assessment of the work in these articles will focus solely on whether the information would be usable to people in both broad and specialist communities, that the work is scientifically sound, and that all associated Research Objects are open, accessible, and follow best (FAIR) practices for sharing [6].

The next step in this iterative pathway will be addressed with an article type we plan to release in the future, namely an Update article. This article type will do two things. First, allow publication of substantive additional data and software versions that are immediately useful to the community but would typically have a much-delayed release or indeed might never be released because the authors need to carry out and add analyses to make these “publishable” in the current publishing paradigm. Second, greatly reduce the time to write and peer-review articles as the majority of the narrative (e.g. the Introduction and similar sections) of an Update does not change in any substantive way and can be linked instead of rewritten, allowing reviewers to focus only on the added information in assessing the manuscript. This type of article is made possible by leveraging the ability of River Valley’s XML-first platform to near instantaneously change and produce content on the fly. Watch this space for more information on this new article type.

## Work Around It or Break Through It

With *GigaScience*, we pushed to break barriers in transparency, reproducibility, and data and tool accessibility: these are our foundational principles. *GigaByte*, on top of having no obstacles to access article text, supporting data and code, plans to break through even more barriers to continue to move toward truly open science. As a medium built for the web rather than one to replace dead trees, *GigaByte* aims to move even further beyond the static PDF. To get there, we are collaborating with River Valley Technologies, whose custom-built hosting platform is flexible and can handle myriad widgets and dynamics. We are encouraging submitting authors to include dynamic features and widgets in their papers to allow greatest interaction with their work. If we are not currently able to do it, we and our partner River Valley Technologies are ready and excited to investigate, explore, and evolve to make this possible. We will, of course, be continuing to carry out open and transparent peer review as standard [7], and peer reviewers will be credited for their hard work with DataCite DOIs that they can display in their online CV and ORCID profiles [8].

Adding to this, we are looking for ways to make the review process more streamlined, to allow reviewers —who all have limited time— to focus primarily on the specific points we want assessed. To do this we will be using a questionnaire style review, but these will also include optional commenting areas so reviewers are not blocked by a static questionnaire from giving their much-desired additional thoughts. Post-publication peer review and further interaction is encouraged through Hypothes.is integration, for collaborative annotation of our content. So readers don’t just have to passively consume information; they can actively connect and update it.

Another barrier to maximising the utility of scientific research to the international community is the lack of focus and tools to aid the reader of the articles. As a clear example, language and jargon issues limit the ability of researchers not fluent in English to engage fully in the international scientific arena and citizen scientists to be able to contribute and provide perspectives not constrained by the current scientific preconceptions. The dynamic River Valley Technologies platform allows integrated language support. It also includes options for readers to select how they want to view papers, including, for example, the availability to view the article in a dyslexic-friendly font. Demonstrating the potential for novel ways of viewing content built on the open XML, we also showcase the open source eLife Lens manuscript viewer, ensuring full use of the highly structured XML files, and allowing you to view relevant content in side-by-side panes [9]. We will be continuing to identify barriers to speed and dynamic presentation in scientific publishing and finding new ways to improve the reader’s interaction with the content.

A final barrier… the cost of publishing. Time is money, as they say, and reducing the time from research to publication is a major goal of ours. But, while the advent of open access publishing has eliminated barriers to accessing information, it has not eliminated the cost of publishing. To be sustainable, open access publishers have moved to having Article Processing Charges (APCs) to cover costs. In moving to open access, publishers have eliminated a major barrier to information, but another barrier has been erected: the ability for some researchers to be able to afford to publish. While we cannot eliminate our costs, we are working hard to make *GigaByte* APCs as low as possible, and certainly *well under* the current mean APC in Europe of €1,975 (∼$2160) [10]. A major cost of doing business in publishing goes into the production of articles, which is typically a manual process to layout and create PDF and XML formats of an article; a lot of this work is still wedded to the print process. The RVT publishing platform has a fully automated production process, enabling us to eliminate that cost and pass that savings to authors. Additionally, our aim is only to cover costs: not to take advantage of grant funds that are aimed at promoting research, and not to make a profit for any business or investor. Following the FAIR Open Access Principles we will also provide transparency on our costs so that authors and granting organizations have a clear understanding of what an APC covers [11]. For authors looking for the greatest savings, that time is now — as there are no APCs for the first 6 months.

## Great fleas have little fleas upon their backs to (Giga)byte ’em

**Figure 2.**
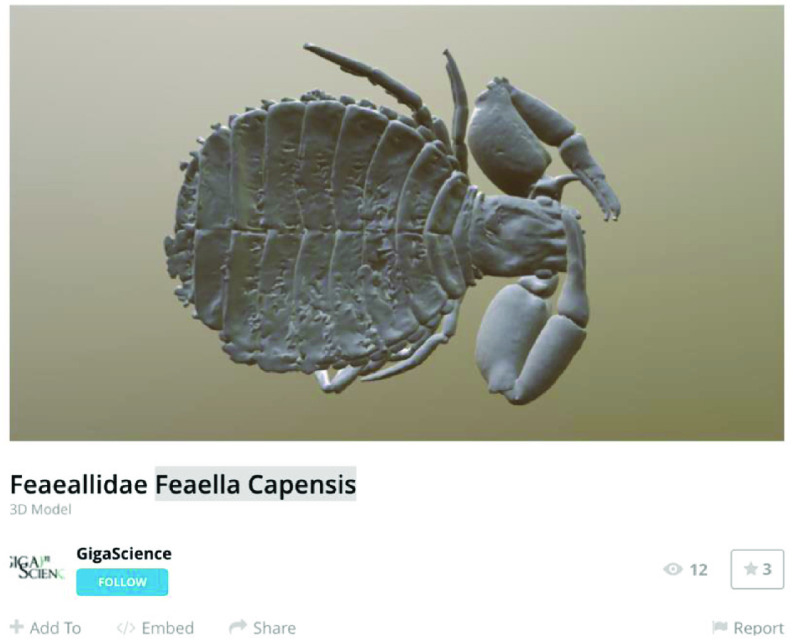
Interactive sketchfab view of pseudoscorpion, *Feaella Capensis*. Data from du Plessis *et al.*, GigaByte, 2020. https://sketchfab.com/3d-models/feaeallidae-feaella-capensis-47e22b7875dc40a49668fe788b5e8af2

With the launch of *GigaByte* and our formal call for papers, we include some articles to give people an idea of the types of functionality our platform provides in addition to the text, and links to data and source code. An example of our new interactive approach to publishing is in our launch Data Release: “Data for 3D Printing Enlarged Museum Specimens for the Visually Impaired”. This presents enlarged museum specimens that were 3D printed for various interactive exhibits at the National Museum in Bloemfontein, South Africa [12]. In describing the data production and re-use potential, the digitised versions of these interactive museum exhibits are equally interactive for *GigaByte* readers, with links to downloadable 3D models in our GigaDB repository [13] and in an embedded Sketchfab window in the article that allows the reader to inspect the model and interact with it through their browser [14]. We also include links to the Thingiverse repository [15] where the 3D printing community can find and download the models and share back their adaptations. Taken from this paper, Figure 2 shows an example of a pseudoscorpion (*Feaella Capensis*). Fun fact: these are also known as ‘book scorpions’ as these tiny arachnids were first described by Aristotle, who likely came across them among scrolls in a library where they would have been feeding on booklice. This article’s data for these book scorpions is a brilliant virtual demonstration of how we have moved well beyond books and scrolls that are prone to an ecosystem of beasts living off them to a digital world that allows us to disseminate, recreate, and remix knowledge — free of barriers such as geography and access (and arachnophobia).

Although digital resources are not prone to booklice, they do have their own risks with “bit rot” and other forms of digital degradation. To mitigate these, we follow best practices, using Crossfef and DataCite digital object identifiers for our textual and data content, and we are members of the CLOCKSS sustainable dark archive to ensure the long-term survival of our textual content. Our integrated GigaDB repository is undergoing CTS certification, and we insist that the data supporting our articles are hosted in trusted data repositories under CC0 public domain waivers.

**Figure 3.**
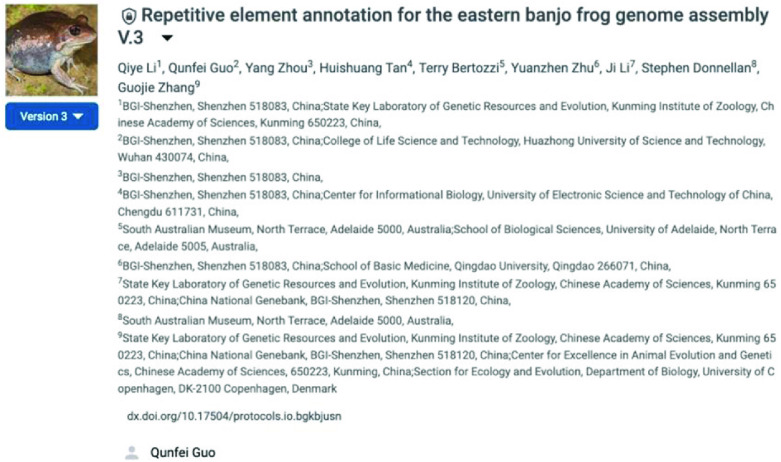
Protocols.io widget for the “repetitive element annotation protocol” http://dx.doi.org/10.17504/protocols.io.bc4niyve

Another area where we’d like to move beyond the textual narrative is how to handle presentation of methods in an article. The traditional “Materials & Methods” format isn’t the best medium for explaining detailed step-by-step methods or complicated computational pipelines. Both computational and wet-lab protocols are much better handled by workflow management systems and protocols repositories. In *GigaScience*, we encourage authors to make use of protocols.io to post their detailed methods and then simply cite that in the article [16]. In *GigaByte*, we are moving to make this even more closely linked to the article. Among our launch articles is a new frog genome [17] demonstrating where the protocols have been integrated into the protocols.io repository (Figure 3) [18].

We hope you enjoy these first exemplar articles, and the innovations seen here are just the start of a process. Working on new infrastructure provides us with a blank canvas to adapt and make changes that were previously heavily constrained by the legacy publishing infrastructure. Future additions to our roadmap include publishing Update articles (noted above), increasing the interactive nature of our papers with more plugins, and bringing in execution and data quality checks in the data and code review process. This will be done both via automated tools and using independent validation through efforts like the CODECHECK certificate of reproducible computation first showcased in *GigaScience* [19].

We encourage you to contact any of the editors to begin conversations about specific needs in your research communities for promoting large-data access, sharing, use, and reuse.

## Abbreviations

APC: Article Processing Charge; CTS: Core Trust Seal; DOI: Digital Object Identifier; FAIR: Findable, Accessible, Interoperable, Re-usable; RVT: River Valley Technologies; XML: Extensible Markup Language.
