# Supplementary material for: GigaByte: Publishing at the Speed of Research
Source: GigaByte. 2020 Jul 1;2020:gigabyte1. doi: 10.46471/gigabyte.1 (PMC9631982; doi:10.46471/gigabyte.1)
Supplement: Translation

# Translation: *GigaByte*: 让发表与研究并驾齐驱

**Authors:** Scott C. Edmunds, Laurie Goodman

**Affiliations:** ^1^ GigaScience, BGI Hong Kong Tech Co Ltd., 26F A Kings Wing Plaza, 1 On Kwan Street, Shek Mun, Sha Tin, NT, Hong Kong, China

## Abstract

随着科技领域日新月异地发展，现行的科学出版方式已无法满足其快速的变化，也不利于展示不断更新的数据集和软件工具。有鉴于此，以及我们对“推动科学出版方式以适应现代研究需求”的不懈追寻，我们很高兴地宣布：一本在线的、完全开放获取、开放数据期刊——*GigaByte*正式创刊。*GigaByte*旨在以一种全新的“软件”模式发表研究成果：代码、版本、派生、更新和重演（CODE、RELEASE、FORK、UPDATE和REPEAT）。继*GigaScience*在促进数据共享和研究的可重复性方面取得成功之后，新创姐妹刊*GigaByte*力求更进一步。*GigaByte*发表的文章篇幅简短，审稿过程采用问卷形式，并将这一审稿形式与River Valley Technologies公司开发的定制版出版基础架构相结合，从而拥有了最先进的可扩展标记语言优先（XML-first）出版平台。该出版平台以整个出版过程更简便、快捷、更具交互性为目标而专门设计，以期与当代的研究交流速度并驾齐驱。

## 回首过去、展望未来

2012年，*GigaScience* [1]创刊，它作为一种新型期刊，将标准的科学出版直接与数据库相关联，存储所有相关数据。为了解决Buckheit和Donoho在1995年提出的问题：“一篇关于算法的文章只是在做广告，而非学术。真正的学术是由完整的软件环境、代码和数据及其共同产生的结果构成的” [2]，我们推出了这一新型期刊，致力于确保在文章中包含各类研究真正的学术成果。

八年来，我们完成了大多数的目标。在获得各大出版商的认可后，数据出版变得越来越主流，期刊也获得了各种奖项（例如，2018年荣获 Prose“期刊出版创新奖” [3]和其他形式的表彰，均是对我们过去努力的认可，我们渴望做的更多。然而，阻碍我们前进的主要因素之一就是传统的出版基础架构，该基础架构的大多数功能仍与印刷生产流程相关联，代码库也十分陈旧，不再适合于非印刷、在线、以数据为中心的数字化时代。在与不断发展的开放科学平台生态系统进行协作和集成时，改变科学出版方式的尝试始终是临时性的，且不得不将其强加在传统出版工作流程中。在技术上更改和更新出版系统通常十分缓慢且有诸多漏洞，并且存在可扩展性问题。此外，更改系统非常昂贵，因此，从商业角度出发，对系统所做的更改必须对很多期刊都有用，虽然所有期刊的需求本就各不相同。现代的、基于网络的平台主导着其他领域，因此，我们应以相同的方式发表现代的、基于网络平台的研究成果。目前，绝大多数出版平台仍依赖于静态的、手工排版PDF，而不是基于现代的、数字优先、基于可扩展标记语言（XML）的技术。

*GigaScience*编辑委员会成员Carole Goble在2013年凭借“Don't Publish. Release！” [4]的提议荣获FORCE11视觉奖，她提议在学术交流中采用21世纪的软件版本模式，而不是18世纪的书籍出版模式。这一提议给我们带来了启发，最终萌生了创立*GigaByte*的想法。

在2015年FORCE11视觉奖的获奖者中，来自River Valley Technologies公司的Kaveh Bazargan提出了“我们为什么如此依附于附件？抛弃它们，改善出版流程” [5]。Kaveh在会上提出了一些初步设想，这些想法最终将成为RVT的端到端出版解决方案，包括投稿、内容管理以及利用合作的在线平台进行托管[图 1]。我们感觉到这正是实现开放科学目标所需要的，也促使我们合作开发了这一出版流程，新的出版平台除了可以即时发布文章外，还将发表可以版本化和派生的交互式文章，从而遵循了“Don't Publish. Release！”的理念。

**图1.** 该录音和幻灯片为2015年1月13日Kaveh Bazargan在牛津FORCE2015“视觉奖”上展示的报告“我们为什么如此依附于附件？抛弃它们，改善出版流程” https://youtu.be/aFzRVqTNi-8

我们与RVT的合作采用新的出版策略和技术，旨在尽最大可能解决上述瓶颈，进一步使我们能够无需对现有的出版流程进行繁琐的变更工作就能改进、微调出版系统，并减少出版时间和成本。

## 3，2，1，创刊！

目前，*GigaByte*将发表两种文章类型：数据释放（*Data Release*）和技术释放（*Technical Release*），分别发表数据集和软件/计算工作流，因为这两类文章是最显而易见需要以迭代形式（代码、版本、派生、更新和重演）发表的研究对象。对这些文章内容的评估将仅关注以下信息：该信息是否可为大众和业界所用；研究是否科学合理；所有相关的研究对象是否公开、可获取且遵循共享 [6]的最佳实践（FAIR）。

下一步要解决的是“以迭代形式发表文章”，未来我们将推出另一个文章类型，即“更新文章”（Update article）。这一类型文章可实现两个目的。第一，允许发表实质性的新增数据和软件更新，这类更新可以立刻为学界所用，但通常会延迟很久发表，甚至可能永远不会发表，因为作者需要进行分析并增加分析以使这些更新在当前的出版模式下“可发表”。第二，大大减少了文章撰写和同行评议的时间，因为更新类文章的大部分描述性内容（例如，引言和类似部分）都没有任何实质性的改变，可以与原始文章相关联而不是重写，从而允许审稿人在审稿过程中仅关注新增信息。利用River Valley的可扩展标记语言优先（XML-first）平台，可以使此类文章实现即时更改和实时生成内容。敬请关注有关该新文章类型的更多信息。

## 变被动迂回为主动出击

研究的透明、可重复以及数据和工具的可获取是*GigaScience*的基本原则，我们已突破这些阻碍。*GigaByte*除了无障碍访问文章、支持性数据和代码外，还将突破更多阻碍，进一步朝着真正开放科学的方向发展。作为网络媒介，而非固有体系的替代品，*GigaByte*旨在超越静态PDF。为了达到这个目标，我们正在与River Valley Technologies合作，该公司的定制托管平台非常灵活，可以植入各种插件和动态系统。我们鼓励作者在文章中加入动态功能和插件，以最大程度地实现互动。对于目前无法实现的功能，我们和我们的合作伙伴River Valley Technologies愿意随时进行研究、探索和改进。当然，我们将继续以进行公开和透明的同行评审为准则 [7]，审稿人的辛苦付出将得到认可，他们可以在在线CV和ORCID个人资料中展示DataCite DOI [8]。

除此之外，我们正在寻找使审稿流程更加简化的方法，即让时间有限的审稿人重点关注我们希望其评议的具体内容。为此，我们将采用问卷形式的审稿方式，但仍会提供可选的评论区域，从而使审稿人不因受限于固定的问卷，而无法表达其他必要的想法。通过整合Hypothes.is，我们鼓励发表后的同行评议及互动。读者可以在文章发表后对文章内容进行注释，不必被动地接受信息，而是可以主动沟通并更新信息。

对国际社会来说，最大限度地利用科学研究的另一个障碍是缺乏关注以及可以帮助读者的工具。举一个明显的例子，语言问题限制了不擅长英文的研究人员全面参与国际科学活动的能力，而专业术语使得公众科学家无法做出贡献并提出不受当前科学先入之见所束缚的观点。动态的River Valley Technologies平台提供了多语言支持，还包括供读者选择阅读方式的选项，例如，可以选择读写障碍友好的字体浏览文章。为了证明以新颖的方式查看基于开放XML的内容的潜力，我们还将展示开源eLife Lens稿件查看器，确保充分利用高度结构化的XML文件，并允许读者在并排窗口中浏览相关内容 [9]。我们将继续寻找各种阻碍，以加速和动态呈现科学出版，并寻找改善读者与内容互动的新方法。

最后一个阻碍——出版费用。正如人们所说，时间就是金钱，减少从研究到发表的时间是我们的主要目标。但是，尽管开放获取出版的出现消除了访问信息的障碍，但并没有消除发表的成本。为了可持续发展，开放获取出版商采用文章处理费（APC）来填补开支。在转向开放获取时，出版商消除了信息访问这一主要障碍，但又建立了另一个障碍：部分研究者是否有能力负担出版费用。虽然我们无法消除成本，但我们仍在努力使*GigaByte*的APC尽可能低，并远低于当前欧洲平均APC水平——1,975欧元（约合2,160美元） [10]。出版过程中的主要业务成本来自于文章的生产（production）环节，这一环节主要是手动排版和创建文章的PDF和XML格式；这项工作中的许多内容仍与印刷流程有关。RVT出版平台具有完全自动化的生产流程，使期刊能够消除该成本，为作者节省该项费用。此外，我们的目标仅是收支平衡：不从旨在促进科学研究的基金中获利，也不为任何企业或投资者赚钱。遵循FAIR的开放获取原则，我们还将公开出版成本，以便作者和资助机构对APC的开支有清晰的了解 [11]。对于需要节省出版费用的作者来说，现在正是发表文章的好时机——因为前6个月本刊将不收取APC。

## 千里之行，始于足下

随着*GigaByte*创刊并正式征稿，我们发表了部分文章。读者们可以籍此了解平台提供的除文本外的功能类型，以及访问链接的数据和源代码。第一篇数据释放（Data Release）文章就展示了新的交互式出版方式：“向视觉障碍人士展示博物馆标本放大模型的三维打印数据”（Data for 3D Printing Enlarged Museum Specimens for the Visually Impaired）。文章展示了通过三维打印放大后的博物馆标本，这些模型是南非布隆方丹国家博物馆的互动展品 [12]。在描述数据产出和再利用的潜力时，这些交互式博物馆展品的数字化版本对于*GigaByte*读者具有同样的可互动性，GigaDB存储库 [13]和文章中嵌入式Sketchfab窗口中提供了可下载三维模型的链接，使读者能够查看模型并通过浏览器进行交互 [14]。我们还提供了Thingiverse存储库的链接 [15]，三维打印爱好者可以在其中查找和下载模型并共享其修改的版本。图 2出自该文章，展示了一种拟蝎（*Feaella Capensis*）三维模型。这些体型较小的蛛形纲动物也被称为“书蝎”，最早是亚里士多德描述的，他很可能是在图书馆的书卷中看到这些以书虱为食的动物。这些拟蝎的数据是虚拟展示的优秀范例，代表着人类超越了兵燹水火、野兽横行的蛮荒时代，进入无视地理障碍和知识获取壁垒（以及蜘蛛恐惧症等困难）地传播、复原和重组知识的数字世界。

**图2.** 拟蝎的交互式sketchfab浏览。数据来源：du Plessis et al., GigaByte, 2020. https://sketchfab.com/3d-models/feaeallidae-feaella-capensis-47e22b7875dc40a49668fe788b5e8af2

尽管数字资源不会被书虱啃噬，但是它们确实存在“数据衰减”和其他形式的数据毁坏风险。为了减少这些问题带来的影响，我们遵循最佳实践，赋予文本和数据内容Crossref和DataCite数字对象标识符（DOI），我们也是CLOCKSS可持续性闭架存储系统的成员，以确保文本内容的长期保藏。GigaDB存储库为CTS认证，我们坚持将文章的支持性数据存储在可信任的数据存储库中，并获得CC0豁免。

我们想要超越文本描述的另一个方向是如何呈现文章中的方法部分。传统的“材料和方法”部分无法将详细的步骤或复杂的计算过程一一展示。工作流管理系统和操作流程存储库可以更好地处理计算过程和湿实验操作流程。对于*GigaScience*，我们鼓励作者使用protocols.io发布其详细实验方法，然后在文章中简单引用 [16]。对于*GigaByte*，我们更是努力推动protocols.io的引用。在我们首批发表的文章中，新的青蛙基因组文章 [17]已将实验方法集成到protocols.io存储库中 [图 3] [18]。

**图3.** 东班卓琴蛙的重复元件注释方法，发布于protocols.io。 http://dx.doi.org/10.17504/protocols.io.bc4niyve

我们希望您喜欢这些示例文章，这里看到的创新仅仅是个开始。新的基础架构为我们提供了一块空白画布，供我们适应和改变，从而摆脱旧的出版基础架构的诸多限制。未来发展路线包括发表更新文章（Update articles，如上所述），使用更多插件增强文章的交互性，以及在数据和代码审查过程中增加管理和数据质量检查。这既可以通过自动化工具来完成，也可以通过使用独立验证来完成，诸如*GigaScience*中首次展示的可重复计算过程的CODECHECK证书等 [19]。

若您的研究团队在促进大数据访问、共享、使用和再利用方面有任何特殊需求，欢迎您联系我们的任何一位编辑咨询。

## 缩略词

APC: Article Processing Charge; CTS: Core Trust Seal; DOI: Digital Object Identifier; FAIR: Findable, Accessible, Interoperable, Re-usable; RVT: River Valley Technologies; XML: Extensible Markup Language.

## 作者贡献

文章撰写: S.C.E., L.G.; 概念形成: S.C.E.

## References

[1] GoodmanL, EdmundsSC, BasfordAT, Large and linked in scientific publishing. Gigascience, 2012; 1(1): doi:10.1186/2047-217X-1-1.PMC361744823587310

[2] BuckheitJB, DonohoDL, WaveLab and Reproducible Research. In: AntoniadisA, OppenheimG (eds), Wavelets and Statistics. New York: Springer 1995; pp. 55–81.

[3] AAP. (2018): 2018 Award Winners - PROSE Awards. https://proseawards.com/winners/2018-award-winners/.

[4] GobleC, (2013): Don’t publish. Release! *FORCE11 Vision Prize Winner* https://www.force11.org/presentation/dont-publish-release.

[5] BazarganK, (2015): Why are we so attached to attachments? Let’s ditch them and improve publishing. *Zeeba TV*. http://zeeba.tv/why-are-we-so-attached-to-attachments-lets-ditch-them-and-improve-publishing/.

[6] WilkinsonMD The FAIR Guiding Principles for scientific data management and stewardship. Sci. Data, 2016; 3: 160018.2697824410.1038/sdata.2016.18PMC4792175

[7] EdmundsSC, Peering into peer-review at GigaScience. Gigascience, 2013; 2(1): 1, doi:10.1186/2047-217X-2-1.23587291PMC3626537

[8] BazarganM, (2018): River Valley implements ORCID to give credit to reviewers. *River Valley Technologies Press Release*. https://rivervalleytechnologies.com/river-valley-implements-orcid-to-give-credit-to-reviewers/.

[9] eLife. (2013): Seeing through the eLife Lens: A new way to view research. *Inside eLife*. Available at: https://elifesciences.org/inside-elife/0414db99/seeing-through-the-elife-lens-a-new-way-to-view-research.

[10] OpenAPC Initiative. OpenAPC. https://treemaps.intact-project.org/apcdata/openapc/.

[11] Fair Open Access Alliance. The Fair Open Access principles. https://www.fairopenaccess.org/the-fair-open-access-principles/.

[12] du PlessisA, ElsJ, le RouxS, TshibalangandaM, PretoriusT, Data for 3D printing enlarged museum specimens for the visually impaired. Gigabyte, 2020; 1: 10.46471/gigabyte.3.PMC963195936824598

[13] du PlessisA, ElsJ, le RouxS, TshibalangandaM, PretoriusT, 3D printing data from enlarged museum specimens. GigaScience Database, 2019; 10.5524/100648.PMC963195936824598

[14] du PlessisA, (2019): Feaeallidae Feaella Capensis - Download Free 3D model by GigaScience (@GigaDB) [47e22b7]. *Sketchfab*. https://sketchfab.com/3d-models/feaeallidae-feaella-capensis-47e22b7875dc40a49668fe788b5e8af2.

[15] du PlessisA, (2019): 3D Museum Specimens - Scorpions, Pseudoscorpions, Birds, Mites by GigaScience. *Thingiverse*. https://www.thingiverse.com/thing:3869332.

[16] TeytelmanL, StoliartchoukA, KindlerL, HurwitzBL, Protocols.io: Virtual Communities for Protocol Development and Discussion. PLOS Biol., 2016; 14: e1002538.2754793810.1371/journal.pbio.1002538PMC4993360

[17] LiQ, GuoQ, ZhouY, TanH, BertozziT, ZhuY, LiJ, DonnellanS, ZhangG, A draft genome assembly of the eastern banjo frog *Limnodynastes dumerilii dumerilii* (Anura: Limnodynastidae). Gigabyte, 2020; 1: 10.46471/gigabyte.2.PMC963200336824594

[18] LiQ (2020): Repetitive element annotation for the eastern banjo frog genome assembly. *protocols.io*. 10.17504/protocols.io.bgkbjusn.

[19] PiccoloSR, LeeTJ, SuhE, HillK, ShinyLearner: A containerized benchmarking tool for machine-learning classification of tabular data. Gigascience, 2020; 9: doi:10.1093/gigascience/giaa026.PMC713198932249316
